# Autosomal recessive Alport syndrome caused by a novel *COL4A4* splice site mutation: a case report

**DOI:** 10.3325/cmj.2019.60.458

**Published:** 2019-10

**Authors:** Petar Šenjug, Tamara Nikuševa Martić, Marija Šenjug Perica, Maja Oroz, Matija Horaček, Martin Ćuk, Slaven Abdović, Danica Galešić Ljubanović

**Affiliations:** 1 Unit of Nephropathology and Electron Microscopy, Department of Pathology and Cytology, Dubrava University Hospital, Zagreb, Croatia; 2 Department of Biology, University of Zagreb School of Medicine, Zagreb, Croatia; 3 Children's Hospital Srebrnjak, Zagreb, Croatia; 4 University Hospital for Infectious Diseases “Dr. Fran Mihaljević,” University of Zagreb School of Medicine, Zagreb, Croatia; 5 Institute of Pathology, University of Zagreb School of Medicine, Zagreb, Croatia; 6 Department of Pediatrics, Children's Hospital Zagreb, Zagreb, Croatia; Šenjug et al: Autosomal recessive Alport syndrome caused by a novel *COL4A4* splice site mutation

Alport syndrome (AS) is a genetically heterogenic, structural disorder of the glomerular basement membrane (GBM) due to the mutation of *COL4A3*, *COL4A4*, or *COL4A5* genes, which clinically presents as progressive hematuric nephritis with ultrastructural changes of the GBM, high tone sensorineural hearing loss, and ocular lesions. About 15% of AS cases have autosomal mutations of *COL4A3* and *COL4A4* genes, including homozygous and compound heterozygous mutations. Here, we present a case of a two-year-old boy with autosomal recessive Alport syndrome (ARAS) caused by a novel c.193-2A>C COL4A4 mutation. The patient had a delayed motor and sensory development coupled with speech and language delay, megalencephaly, hematuria and proteinuria, and normal tonal audiogram and ophthalmology exam. Extensive genetic, metabolic, and neurologic workup performed at the age of 10 months was unremarkable and patient's megalencephaly was described as familial benign megalencephaly. Kidney biopsy analysis showed characteristic signs of AS. Mutations screening with use of Illumina MiSeq platform revealed that the patient was homozygous for a newly discovered splice acceptor pathogenic variant c.193-2A>C found in *COL4A4* at the genomic position chr2:227985866 and both parents were heterozygous carriers. The genetic heterogeneity of AS makes the diagnostic process challenging. Although renal biopsy provides information about the characteristic GBM changes and the degree of renal parenchyma damage (interstitial fibrosis and tubular atrophy ratio), genetic testing is a more sensitive and specific method that also gives insight into disease severity and clinical course, and provides the basis for genetic counseling.

Alport syndrome (AS) is a genetically heterogenic disorder that clinically presents as a progressive nephropathy characterized by hematuric nephritis with ultrastructural changes of the glomerular basement membrane (GBM), high tone sensorineural hearing loss, and ocular lesions (1). The genetic base for AS is most frequently (85%) the mutation of X-linked gene *COL4A5* (2). Only about 15% of AS cases have autosomal mutations of *COL4A3* and *COL4A4* genes, including homozygous and compound heterozygous mutations (3). X-linked AS more often and more severely affects men than women (4). However, in autosomal recessive AS, the clinical course is influenced by the genotype but is usually severe in both men and women, with early progression to end stage renal disease (ESRD) and frequent extrarenal manifestations (5). Here, we present a case of an early onset ARAS with characteristic GBM changes caused by a novel, c.193-2A>C COL4A4 mutation.

## Case report

**Figure 1:**
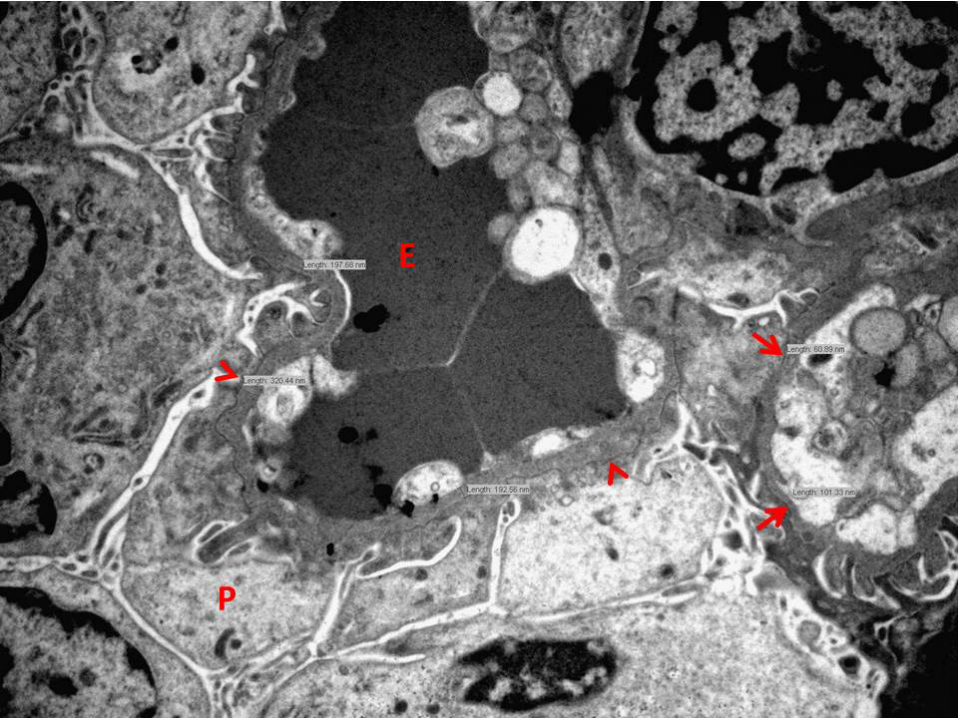
Thinning (arrows) and thickening (arrow heads) of glomerular basement membrane with lamellation in the areas of thickening. P – podocyte, E – erythrocyte. Electron microscopy, magnification ×8000.

A 10-month-old male infant was referred to the Department of Pediatrics, Children's Hospital Zagreb in 2016 due to a delay in the psycho-motoric development and megalencephaly. After extensive genetic, metabolic, cytogenetic, and neurologic workup, clinical phenotype of the child was described as familial benign megalencephaly. The patient had both motor and sensory developmental delay, coupled with speech and language delay, with stereotypical movements, unresponsiveness to name calling at the age of one, lack of sentence formation with a limited number of used words for age, short attention span, bad tolerance to frustration, and the onset of walking at 22 months. At the age of 1 year and 2 months, macrohematuria and proteinuria were recorded for the first time, with proteine/creatinine ratio of 284 mg/mmol. At the age of 2 years he had proteinuiria 189 mg/mmol and severe microhematuria. Tonal audiogram was unremarkable and eye exam did not reveal anterior lenticonus. Kidney biopsy was performed. Light microscopy analysis showed one out of 69 globally sclerosed glomeruli and 30% of immature/partly immature glomeruli. There was one small focus of interstitial fibrosis and tubular atrophy (IFTA) that affected 1% of the cortical parenchyma. Routine immunofluorescent analysis was unremarkable. Electron microscopy (EM) of the GBM showed areas of thinning and thickening (61 to 390 nm, average 167 nm with a standard deviation of 65 nm, while the reference range of GBM thickness for the age of 2 years is 156-292 nm, Figure 1) (6). The thickened areas of GBM exhibited lamellation and basket-wave appearance. There was focal podocyte foot process effacement in the areas above the thickened and lamellated basement membranes. Kidney biopsy report concluded that the findings corresponded to AS and suggested genetic counseling and testing.

Ramipril treatment (6 mg/m^2^) reduced proteinuria to 73 mg/mmol creatinine, while severe microhematuria persisted.

**Figure 2:**
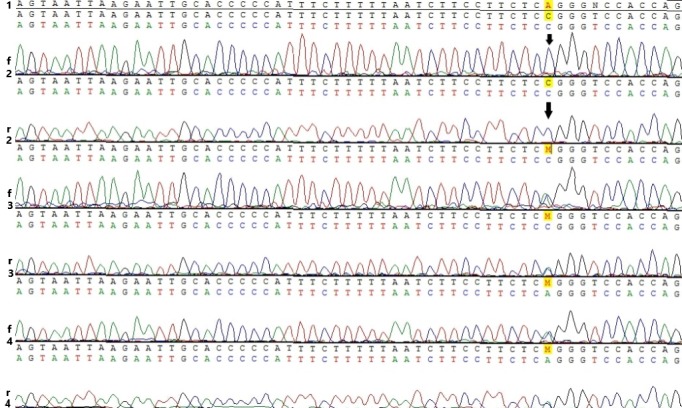
Mutation analysis of c.193-2A>C in *COL4A4* at the genomic position chr2: 227985866. Electropherogram of a single base-pair A>C substitution at nucleotide 193 (marked with yellow color) in an affected patient [2] and carrier parents [3 and 4]. Patient's and parents’ electropherograms are presented as bidirectional sequences (forward [f] and reverse direction [r]). The sequence numbered as [1] stands for reference *COL4A4* sequence. M stands for mixed bases (heterozygous genotype) of A and C.

*COL4A3*, *COL4A4*, and *COL4A5* genes of both the patient and his parents were screened for mutations with use of Illumina MiSeq platform (Illumina, San Diego, CA, USA). For bioinformatical analysis, Illumina VariantStudio software was used. All variants were assigned a number in available databases, including the NCBI dbSNP138 and ClinVar (7). Newly discovered splice acceptor pathogenic variant c.193-2A>C found in *COL4A4* at the genomic position chr2:227985866 (variant described according to reference genome GRCh37) was confirmed with standard dye-terminator sequencing with use of ABI310 (Applied Biosystems, Foster City, CA, USA) with BigDye v1.1 and Vector NTI Software (Thermo Fisher Scientific, Waltham, MA, USA) for visualization. Both parents were heterozygous for c.193-2A>C variant, while the patient was homozygous (Table 1) (Figure 2).

**Table 1:** Medical history timeline

Year/age	Symptoms	Diagnostic workup	Diagnosis	Therapeutic intervention
February 2016/0 months	APGAR score 10/10, weight 3090 g, length 48 cm			
December 2016/10 months	Delay in the psycho-motoric development and megalencephaly	Cariogram 46 XY, tests for fragile X, organic acids in urine, homocysteine B12 and folic acid, acyl – carnitine profile, amino acids in urine and serum	Familial benign megalencephaly	
April 2017/14 months	Macrohematuria and proteinuria	Protein/creatinine ratio of 284 mg/mmol		
February 2018/24 months	Proteinuria and severe microhematuria	Protein/creatinine ratio of 189 mg/mmol		Ramipril treatment with proteinuria decrease
		Kidney biopsy	Alport syndrome	
		Tonal audiogram	Normal	
		Ophthalmology exam	Normal	
August 2018/30 months		Genetic testing	c.193-2A>C COL4A4 mutation	

## Discussion

The pathohistological diagnosis of AS in children can be challenging. EM in children with AS often shows only thin glomerular basement membranes (5). In our patient, light microscopy analysis showed 70% of glomeruli with normal appearance, one globally sclerosed glomerulus, and 30% of glomeruli with immature/partly immature appearance. This percentage, although somewhat above the expected for the patient’s age, is usual in AS patients (8,9). There was one small focus of IFTA, which affected 1% of the cortical parenchyma. EM revealed classic AS ultrastructural morphology.

Genetic analysis in our patient revealed splice site c.193-2A>C in *COL4A4* at the genomic position chr2:227985866. This mutation has not been previously described in either The Human Gene Mutation Database, Leiden Open (source) Variation Database, or Ensembl genome database. The mutation met the PVS1, PM2, and PP3 criteria for pathogenicity by American College of Medical Genetics and Genomics (10). It was a null variant (within ±2 of canonical splice site) affecting *COL4A4* gene, which is a widely known disease mechanism (PVS1); the allele was not found in GnomAD despite good coverage (PM2); and there was computational evidence of pathogenicity (PP3) with DANN score: 0.9909; GERP scores: NR 5.2399 and RS 5.2399; MutationTaster: accuracy 1 and coverted rankscore 0.8103; dbscSNV: ADA score 0.9999 and RF score 0.916. Variant comparison to the in-house database of 50 healthy individuals also supported this finding.

Very few potential splicing mutations are identified within the first 10 nucleotides of the intron-exon boundaries for the *COL4A3* and *COL4A4* genes (11). In our case, we observed the mutation c.193-2A>C in *COL4A4* at the genomic position chr2:227985866. There are reports of *COL4A4* splice site mutation causing autosomal AS (12). Rosado et al (12) found IVS3 + 1G>C, replacement of guanine to cytosine in position 1+ of intron 3 in the splicing region, suggesting the presence of autosomal dominant AS. In our case, patient’s both parents were heterozygous carriers of c.193-2A>C. The first testing showed that the parents had no hematuria or proteinuria and had normal kidney function, while a repeated urine analysis showed that the father had hematuria. The patient was homozygous for c.193-2A>C, suggesting autosomal recessive inheritance. Parents denied consanguinity, however they were both born in the same village, indicating a theoretical possibility of consanguinity.

It is hard to predict the clinical course for our patient. On the kidney biopsy level, light microscopy showed characteristic EM changes and only incipient chronic changes. Savige et al (11) reported that patients with *COL4A4* mutation develop end-stage renal failure at a mean age of 25.4 ± 10.3 years. Our patient at the age of two had proteinuria and hematuria with normal kidney function.

Considering the genetic heterogeneity of the disease, diagnostic process of AS can be challenging. We presented a case of ARAS caused by a novel c.193-2A>C COL4A4 splice site mutation. Although renal biopsy provides information about the degree of renal parenchyma damage, genetic testing is a more sensitive and specific method that also gives insight into potential disease severity and clinical course, and provides a basis for genetic counseling.
